# Correction: A content analysis of Canadian influencer crisis messages on Instagram and the public’s response during COVID-19

**DOI:** 10.1186/s12889-022-14203-8

**Published:** 2022-10-03

**Authors:** Melissa MacKay, Caitlin Ford, Taylor Colangeli, Daniel Gillis, Jennifer E. McWhirter, Andrew Papadopoulos

**Affiliations:** 1 Department of Population Medicine, University of Guelph, Guelph, ON N1G2W1 Canada; 2 School of Computer Science, University of Guelph, Guelph, ON N1G2W1 Canada

**Correction: BMC Public Health 22, 763 (2022)**

**https://doi.org/10.1186/s12889-022-13129-5**

In the original publication of this article there was an error in some of the excel formulas and they pulled from the wrong data.

This caused errors in the below locations:

- Result section
- Discussion section
- Table 5, 6 and 7

This doesn’t have a large impact on the results/conclusions, as the cell values were correct, it was the reporting of the percentages. There was no real consistency among the values across the various influencer types and their use in the captions and images, so the interpretation of the results remain the same.

The original article has been updated to correct these errors. The affected sections are shown in Additional file 1 (yellow highlight).

## Supplementary Information

**Additional file 1: Supplementary file 1.**

